# A lateral flow assay for the immunodiagnosis of human cat‐transmitted sporotrichosis

**DOI:** 10.1111/myc.13516

**Published:** 2022-08-25

**Authors:** Regielly Cognialli, Konner Bloss, Izabella Weiss, Diego H. Caceres, Rachelle Davis, Flavio Queiroz‐Telles

**Affiliations:** ^1^ Mycology Unit, Hospital de Clínicas Federal University of Paraná Curitiba Brazil; ^2^ Postgraduate Program in Internal Medicine and Health Science Federal University of Paraná Curitiba Brazil; ^3^ Immuno‐Mycologics (IMMY) Norman Oklahoma USA; ^4^ Department of Clinical Analysis Federal University of Paraná Curitiba Brazil; ^5^ Center of Expertise in Mycology Radboudumc/CWZ Nijmegen The Netherlands; ^6^ Studies in Translational Microbiology and Emerging Diseases (MICROS) Research Group, School of Medicine and Health Sciences Universidad del Rosario Bogota Colombia; ^7^ Department of Public Health, Hospital de Clínicas Federal University of Paraná Curitiba Brazil

**Keywords:** antibody, Cat‐Transmitted Sporotrichosis, immunodiagnosis, lateral flow assay, mycosis, *Sporothrix brasiliensis*, sporotrichosis

## Abstract

**Background:**

Cat‐transmitted sporotrichosis (CTS) caused by *Sporothrix brasiliensis* has emerged as an important zoonosis in Brazil and neighbouring countries.

**Objectives:**

Evaluate the performance of a lateral flow assay (LFA) for the detection of anti‐*Sporothrix* antibodies in human sera.

**Methods:**

A LFA for the detection of anti‐*Sporothrix* antibodies (Anti‐Sporo LFA) in human sera, developed by IMMY, was evaluated using 300 human sera collected prospectively at the Hospital de Clínicas, Federal University of Paraná (HC‐UFPR), in Curitiba, Brazil. These specimens included 100 sera from patients with CTS. CTS cases were classified as follows: 59 lymphocutaneous, 27 fixed cutaneous,13 ocular, and one mixed form. One‐hundred specimens from patients with other mycoses, including cryptococcosis (*n* = 32), candidemia (*n* = 27), paracoccidioidomycosis (*n* = 14), aspergillosis (*n* = 10), histoplasmosis (*n* = 9), fusariosis (*n* = 4), lobomycosis (*n* = 1), chromoblastomycosis (*n* = 1), mucormycosis (*n* = 1) and *trichosporonosis* (*n = 1*). And 100 specimens from apparently healthy volunteers (AHV).

**Results:**

The Anti‐Sporo LFA showed a global sensitivity of 83% (95% confidence interval [CI] = 74%–90%), a global specificity of 82% (95% CI = 76%–87%), and accuracy of 82% (95% CI = 77%–86%). By clinical form sensitivity was as follows: Mixed form 100%, ocular 92%, lymphocutaneous 83% and fixed cutaneous 78%. False‐positive results were observed in 11 specimens from people with other mycoses and 26 specimens from AHV.

**Conclusion and discussion:**

This study presents the results of the evaluation of the first lateral flow assay for the detection of anti‐*Sporothrix* antibodies in human sera. The findings here show evidence that IMMY's Anti‐Sporo LFA is a promising tool for the rapid diagnosis of CTS.

## 1 INTRODUCTION

Sporotrichosis is a neglected implantation mycosis caused by *Sporothrix* spp. This is a disease with global distribution, but it is more frequently reported in tropical and subtropical regions around the world.^1^, ^2^, ^3^, ^4^, ^5^ Sporotrichosis has an estimated global annual incidence rate of more than 40,000 cases per year, mainly located in endemic areas, but the data are limited due to the lack of mandatory reporting of this disease in most of the affected countries.^6^

Since the late 1990s, an outbreak of cat‐transmitted sporotrichosis (CTS) emerged initially in the state of Rio de Janeiro, Brazil. Then, in 2007, a new species, *S. brasiliensis*, was identified using molecular analysis.^1^, ^7^, ^8^, ^9^ This species has the capacity of zoonotic transmission and emerged as an agent of cat‐transmitted sporotrichosis (CTS).^1^, ^7^, ^8^ In the last two decades, *S. brasiliensis* has spread, causing CTS outbreaks across the Brazilian territory and in recent years, sporadic cases in Argentina, and Paraguay.^2^, ^5^, ^8^, ^10^, ^11^, ^12^, ^13^, ^14^, ^15^, ^16^

A sick domestic cat (*Felis catus*) transmits the yeast form of *S. brasiliensis* to other cats, humans, and dogs. Infection occurs by traumatic implantation largely from scratching and biting, and in some cases, by cutaneous or mucosal implantation by contact with skin exudates and respiratory secretions from sick cats.^1^, ^2^ *Sporothrix brasiliensis* is more virulent than other species of the *Sporothrix* genus, and the fixed lymphocutaneous and cutaneous forms are the most frequent clinical forms. However, CTS epidemic atypical clinical forms have been described as ocular, antigen hyperreactivity, osteoarticular, meningitis and other extracutaneous infections, adding challenges to the diagnosis of this disease.^1^, ^2^, ^3^, ^5^, ^7^, ^17^, ^18^, ^19^

Due to the broad clinical spectrum of this disease, the differential diagnosis of sporotrichosis with other infectious and non‐infectious diseases must be carried out, including tegumentary leishmaniasis, tuberculosis, pyoderma, cat scratch disease, chromoblastomycosis, phaeohyphomycosis and mycetoma.^5^ Due to the low specificity of the clinical manifestation, the use of specific laboratory assays is key.^1^, ^20^

To control the zoonotic spread of this disease, the implementation of the One Health approach is needed. This approach integrates human, animal and environmental professionals such as microbiologists, veterinarians, physicians, epidemiologists and surveillance officers.^8^, ^16^, ^21^ One of the key aspects of human and animal patients with zoonotic sporotrichosis is its early detection.^16^, ^22^ In addition, due to the increase of CTS with atypical manifestations, it is necessary to implement rapid and accurate testing.^1^, ^2^, ^16^, ^18^

The standard method for the diagnosis of sporotrichosis is the isolation of the fungus by culture, but this method is time‐consuming, requires well‐trained professionals, and has variable sensitivity.^1^, ^20^, ^23^, ^24^ Direct examination and histopathological study have low sensitivity; less than 30%.^8^, ^20^ Intradermal reactivity using sporotrichin is not commercially available and its use is limited to some few highly specialised medical centres.^20^, ^23^ For the immune diagnosis of sporotrichosis, there is a commercially available latex agglutination system for antibody (Ab) detection, but its analytical performance varies according to the clinical form, ranging from 100% sensitivity for the diagnosis of disseminated forms, to 56% for the diagnosis of cutaneous disease.^8^, ^20^ Some in‐house enzyme immunoassays (EIA) have been developed, showing high sensitivity and specificity, but these assays are limited to just a few laboratories.^8^, ^23^, ^24^

CTS caused by *Sporothrix brasiliensis* is an emerging health problem in Brazil and a threat to bordering countries.^1^, ^7^, ^8^ The aim of this study was to evaluate the performance of a lateral flow assay (LFA) for the rapid detection of anti‐*Sporothrix* antibodies in human sera.

## 2 MATERIALS AND METHODS

### 2.1 Study design

A prospective cross‐sectional study was done. Sera specimens were collected between November 2018 and March 2021 at the mycology laboratory of the Hospital de Clínicas of Federal University of Paraná (HC‐UFPR) in Curitiba, Brazil. Specimens were aliquoted and frozen at −20°C until testing. We included cases with proven and probable CTS, independently of gender, age and clinical form. Patients with more than 30 days of CTS treatment were excluded, and patients classified as possible CTS were also excluded. All study participants were enrolled in the study after accepting their participation by signing the informed consent.

CTS was defined following the guidelines of the Brazilian ministry of health (Appendix A).^24^, ^25^ Mycological studies were done with those patients where access to specimens was possible. All CTS cases were treated and followed during their time at the HC‐UFPR. Cases were not enrolled if they had been treated for CTS for 31 days or more.^25^

### 2.2 Specimens

**FIGURE 1:**
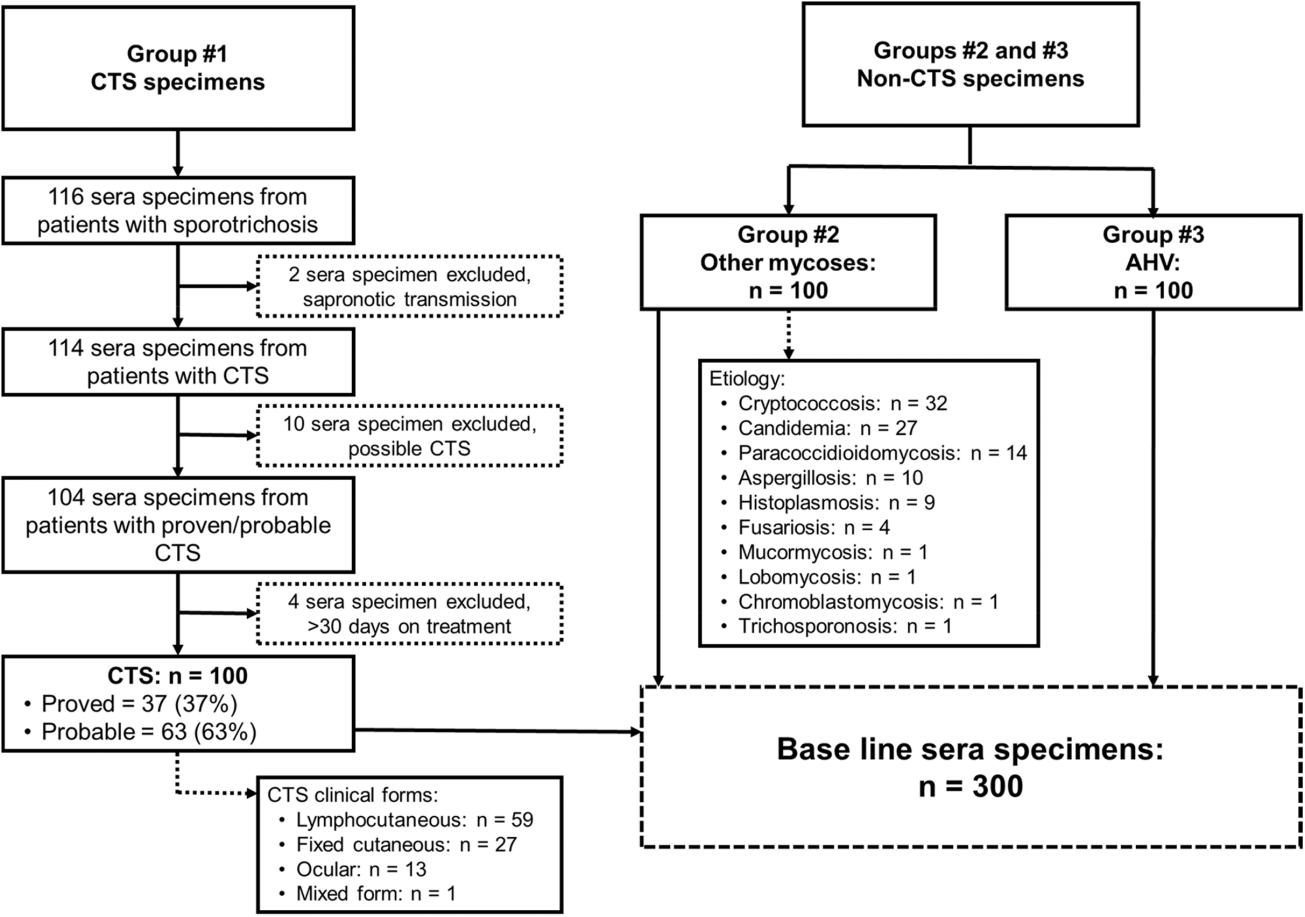
Flow chart of specimens analysed during the evaluation of the performance of the IMMY lateral flow assay for detection of anti‐*Sporothrix* antibodies. Legend: (CTS) cat‐transmitted sporotrichosis; (AHV) apparently healthy volunteers.

A total of 300 human sera specimens were tested. Specimens were classified into the following three groups. Group #1: 100 sera from patients with proven (*n* = 37) or probable (*n* = 63) CTS. By CTS clinical form, we tested 59 patients with lymphocutaneous CTS, 27 patients with fixed cutaneous CTS, 13 patients with ocular CTS, and one patient with mixed form of CTS (osteoarticular and fixed cutaneous). Group #2: 100 sera from patients with other mycoses. This group includes 32 patients with cryptococcosis, 27 patients with candidemia, 14 patients with paracoccidioidomycosis, 10 patients with aspergillosis, nine patients with histoplasmosis, four patients with fusariosis, one patient with lobomycosis, one patient with chromoblastomycosis, one patient with mucormycosis and one patient with t*richosporonosis*. Group #3 corresponded to 100 sera from apparently healthy volunteers (AHV) without contact with sick cats or any lesions (Figure 1).

### 2.3 Lateral flow assay for detection of anti‐*Sporothrix* antibodies

A Lateral flow assay (LFA) for the detection of anti‐*Sporothrix* antibodies (Anti‐Sporo LFA) was developed by IMMY (Norman, OK, USA), and specimens were tested at the mycology laboratory of the HC‐UFPR, in Curitiba, Brazil. The Anti‐Sporo LFA is a nitrocellulose immunochromatographic assay for the detection of antibodies against *Sporothrix* in human serum. This LFA uses a gold conjugate mix of proteins G and L in the sample pad, and in the test line a purified *Sporothrix* antigen, obtained from culture filtrate from mycelial phase, composed of a 50:50 mix of *S. schenkii* (ATCC 58251, https://www.atcc.org/products/58251) and *S. brasiliensis* (ATCC‐MYA 4824, https://www.atcc.org/products/mya‐4824). The control line was a goat anti‐human IgG/IgM.

### 2.4 Specimen preparation and testing

**FIGURE 2:**
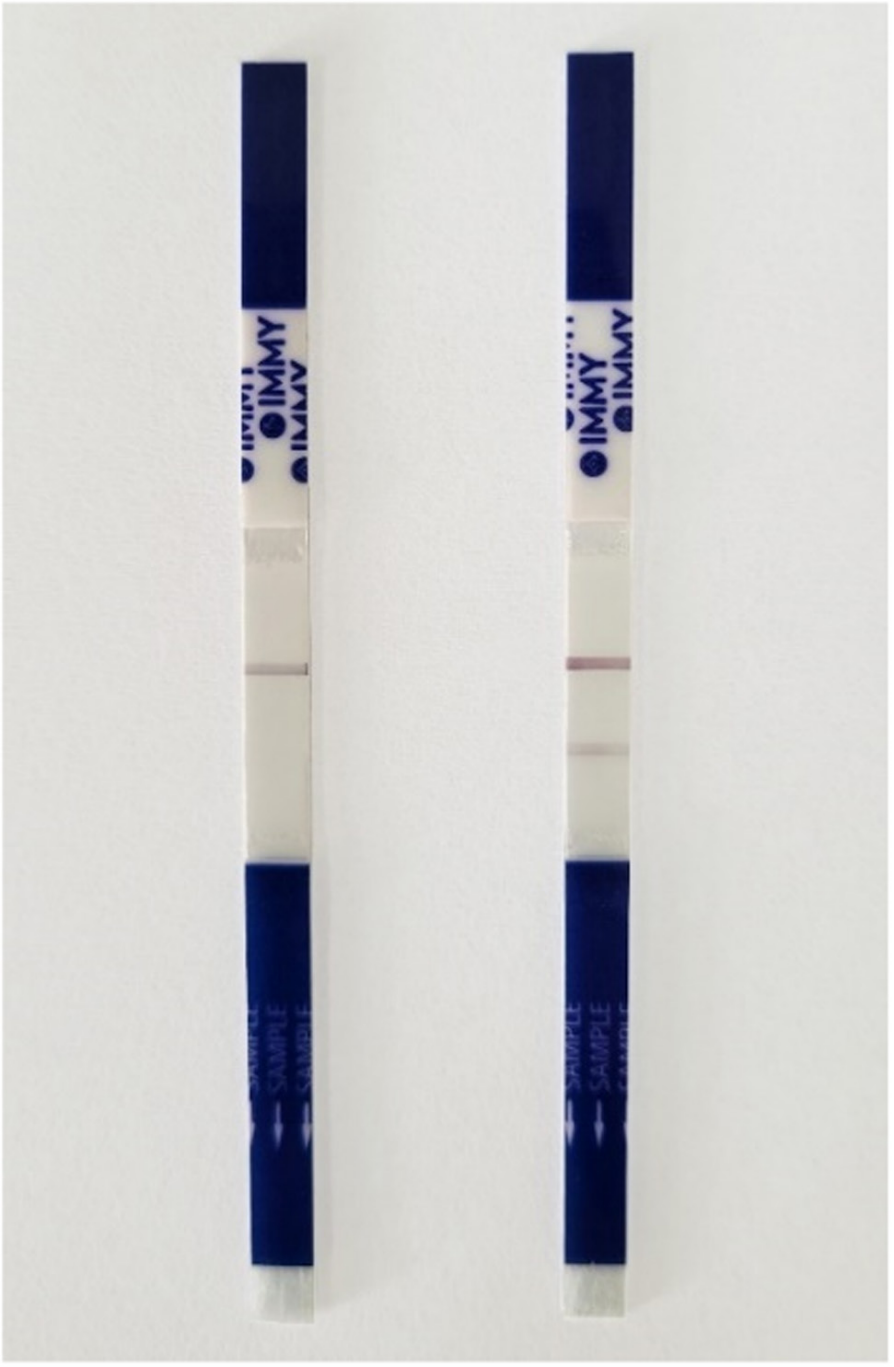
Interpretation of Anti‐Sporo LFA results. Left: A negative LFA, interpreted by only the presence of the control line. Right: A positive LFA, result isinterpreted by the presence of two lines; the control line (top), and the test line (bottom).

First, sera specimens were diluted 1:441 using the kit specimen diluent. 100 μl of the diluted sera was then dispensed into a flat bottom tube/well, followed by the LFA strip. The assay was incubated at room temperature (15–25°C) for 30 min. After 30 min of incubation, the test was interpreted by a visual read. This read was performed by two operators within 10 min after the time of incubation. For results interpretation, the presence of no lines or a test line in the absence of a control line was interpreted as invalid results. Positive results were interpreted as the presence of two lines, a test line and a control line. A negative result was interpreted as the presence of the control line alone (Figure 2).

### 2.5 Statistical analysis

Calculation of the analytical performance of the test was done using 2 × 2 tables comparing the Anti‐Sporo LFA against CTS diagnosis. Using this table, the tests sensitivity, specificity, accuracy, positive and negative predictive values, and their respective 95% confidence intervals (CI) were calculated.^26^ Categorical variables were compared using a chi‐square test with significance level of 5%. Analyses were conducted using MedCalc software.

### 2.6 Ethics statements

This study was approved by the HC‐UFPR Research Ethics Committee under registration CAAE 12379819.4.0000.0096.

## 3 RESULTS

All CTS cases and patients from the control group came from the Curitiba metropolitan area, at the Parana state in Brazil. Among the group of patients with CTS, 116 subjects with sporotrichosis were identified. Of these, two were excluded due to sapronotic infection. Another 10 patients were excluded because CTS was classified as possible. Additionally, four individuals were excluded because they were on antifungal treatment for more than 30 days (Figure 1). We observed a higher prevalence of CTS in women, 1.6:1 sex ratio (62 women and 38 men). In this study, we found 16% prevalence of childhood CTS. All proven CTS were confirmed by molecular identification of *S. brasiliensis*. In the group of 63 probable CTS, five patients were tested by microscopy and culture. All five patients tested negative with both tests. All patients with a diagnosis of CTS received systemic antifungal treatment with itraconazole, terbinafine or both medicines. All cases were cured by the end of treatment.

Using the Anti‐Sporo LFA, we observed a global sensitivity of 83% (95% CI = 74%–90%), a specificity of 82% (95% CI = 76%–87%), positive predictive value of 69% (95% CI = 62%–75%), negative predictive value of 90% (95% CI = 85%–93%) and an accuracy of 82% (95% CI =77–86%) (Table 1).

**TABLE 1:** Analytical performance of the IMMY anti‐*Sporothrix* antibodies LFA

		CTS diagnosis	
		+	−
**LFA**	+	83	37
	−	17	163

	% (95% CI)
Sensitivity	83 (74–90)
Specificity	82 (76–87)
Accuracy	82 (77–86)
Positive predictive value	69 (62–75)
Negative predictive value	90 (86–93)

Abbreviations: −, Negative; +, Positive; 95% CI, 95% confidence interval; CTS, Cat‐transmitted sporotrichosis; LFA, Lateral flow assay.

The Anti‐Sporo LFA correctly classified 83 out of the 100 CTS cases as positive. We observed 86% sensitivity in proven CTS and 81% sensitivity in probable CTS (*p* = 0.477). In the true positives results, *n* = 83 sera, 32 were classified as proven CTS (39%) and 51 probable CTS (61%). In this group of specimens, 49 were from lymphocutaneous CTS, 21 were fixed cutaneous CTS, 12 were ocular CTS and 1 mixed CTS. Performance of the sensitivity of the Anti‐Sporo LFA by clinical forms was lymphocutaneous 83% (95% CI = 71%–92%), fixed cutaneous 78% (95% CI = 58%–91%), ocular 92% (95% CI = 64%–99%) and mixed form 100% (95% CI = 3%–100%) (Table 2). False‐negative results were observed in 17 CTS specimens, 14 female patients (82%) and three male patients (18%). These patients presented a mean age of 51 years (range: 10–80 years old). CTS was defined as proven in 5 patients (29%), and probable in 12 patients (71%). By clinical form, false‐negative results were observed in 6 patients with fixed cutaneous CTS, 10 patients with lymphocutaneous CTS, and one patient with ocular CTS (Table 3). We observed that the sensitivity in children was 88%, and in adults, it was 84%. These were higher in comparison with the group of patients older than 60 years, 75% sensitivity, but these differences were not statically significant (*p* = 0.610) (Table 4). We observed statistically significant differences (*p* = <0.001) in the sensitivity of the Anti‐Sporo LFA when disease onset was more than 21 days (91%), in comparison with patients with early disease, less than 21 days (31%) (Table 4).

**TABLE 2:** Analytical performance of the IMMY anti‐*Sporothrix* antibodies LFA: analysis by clinical form

Clinical form	Sensitivity (95% CI)	Accuracy (95% CI)
Lymphocutaneous (*n* = 59)	83 (71–92)	82 (77–86)
Fixed cutaneous (*n* = 27)	78 (58–91)	81 (75–86)
Ocular (*n* = 13)	92 (64–99)	82 (76–87)
Mixed form (*n* = 1)	100 (3–100)	82 (76–87)

Abbreviations: 95% CI, 95% confidence interval; *n*, Number.

**TABLE 3:** Characteristics of 17 patients with false‐negative results using the IMMY anti‐*Sporothrix* antibodies LFA

	CTS classification	Clinical form	Additional Comments
1	Probable	Fixed cutaneous	♀ 54 YO. DO: one month. WCo. Ulcerated lesion on right hand
2	Proven	Fixed cutaneous	♀ 70 YO. DO: 15 days. WCo. Ulcerated lesion on the back of right hand. *S. brasiliensis* isolated from hand tissue
3	Probable	Fixed cutaneous	♂ 54 YO. DO: 20 days. Hypertension and gastritis. Ulcerated lesion on the right hand
4	Probable	Fixed cutaneous	♀ 56 YO. DO: one month. WCo. Ulcerated lesion on left hand
5	Probable	Fixed cutaneous	♀ 40 YO. DO: 10 days. WCo, veterinary. Papular lesion on right hand
6	Proven	Fixed cutaneous	♀ 56 YO. DO: 14 days. HIV+. Ulcerated lesion on right hand. *S. brasiliensis* isolated from hand exudate
7	Probable	Lymphocutaneous	♀ 27 YO. DO: three months. WCo, veterinary. Lymphangitis
8	Proven	Lymphocutaneous	♀ 36 YO. DO: one month. WCo. Lymphangitis, *S. brasiliensis* isolated from finger tissue
9	Probable	Lymphocutaneous	♀ 51 YO. DO: two months. WCo. Lymphangitis
10	Probable	Lymphocutaneous	♂ 14 YO. DO: 20 days. WCo. Lymphangitis
11	Proven	Lymphocutaneous	♀ 10 YO. DO: two months. WCo. *S. brasiliensis* isolated from hand exudate and lymphangitis
12	Probable	Lymphocutaneous	♀ 60 YO. DO: 21 days. WCo. Nodules on upper and lower limbs
13	Probable	Lymphocutaneous	♀ 21 YO. DO: 15 days. WCo, veterinary student. Nodules on left forearm
14	Proven	Lymphocutaneous	♂ 80 YO. DO: two months. *S. brasiliensis* isolated from arm tissue
15	Probable	Lymphocutaneous	♀ 65 YO. DO: one month. WCo. Nodules on upper and lower limbs and lymphangitis
16	Probable	Lymphocutaneous	♀ 47 YO. DO: 21 days. WCo. Ulcerated lesion on nose and lymphangitis
17	Probable	Ocular	♀ 23 YO. DO: 10 days. WCo. Granulomatous conjunctivitis

*Note*: CTS based Brazilian ministry of health case definitions^24^, ^25^

Abbreviations: −, Negative; ♀, Female; ♂, Male; DO, disease onset; WCo, Without comorbidities; YO, Years old.

**TABLE 4:** Sensitivity of the IMMY anti‐*Sporothrix* antibodies LFA: analysis by group of age and disease onset

	Sensitivity (95% CI)	*p*
Age		
<18 years old (*n* = 16)	88 (62–98)	0.610
19–59 years old (*n* = 68)	84 (73–92)	
>60 years old (*n* = 16)	75 (48–93)	
Disease onset		
<21 days (*n* = 13)	31 (9–61)	<0.001
>21 days (*n* = 87)	91 (83–96)	

*Note*: *p* < 0.001 statistically significant differences.

Abbreviations: 95% CI, 95% confidence interval; *n*, Number.

False‐positive results were found in 37 out of the 200 (18%) non‐CTS specimens. Among these false‐positive results, 11 were from the group of patients with other mycoses (Group #2). We observed that the false positives were cross‐reactions with specimens from patients with histoplasmosis (2 out of 10, 20% cross‐reactivity), candidemia (4 out of 27, 15% cross‐reactivity), paracoccidioidomyocis (2 out of 14, 14% cross‐reactivity), cryptococcosis (2 out of 32, 6% cross‐reactivity) and trichosporonosis (1 out of 1, 100% cross‐reactivity) (Tables 5, 6). The other 26 false‐positive results were observed in specimens from AHV (Group #3). All AHV declared not having comorbidities, contact with sick cats suspected of CTS, or having any previous presentation of symptoms suggestive of sporotrichosis. These subjects were individuals with ages ranging from 14 to 65 years old and were students or healthcare workers. All were residents in the endemic region for CTS. LFA bands in the test zone observed in Group #3 were less intense than bands observed from specimens in Groups #1 and #2.

**TABLE 5:** Summary of cross‐reactivity using the anti‐*Sporothrix* detection antibody LFA

Specimen classification	False positives % (number)
Group 2	
Histoplasmosis	20 (2/10)
Candidemia	15 (4/27)
Paracoccodioidomycosis	14 (2/14)
Cryptococcosis	6 (2/32)
*Trichosporonosis*	100 (1/1)
Aspergillosis	0 (0/10)
Fusariosis	0 (0/4)
Lobomycosis	0 (0/1)
Chromoblastomycosis	0 (0/1)
Mucormycosis	0 (0/1)
Overall cross‐reactivity	11 (11/100)
Group 3	
Apparently healthy volunteers	26 (26/100)

**TABLE 6:** Characteristics of patients in Group #2 with false‐positive results

	Diagnosis	Comments
1	Histoplasmosis	Immunodeficient. Positive histopathology, histoplasmosis
2	Histoplasmosis	Positive culture, *H. capsulatum*
3	Candidemia	*Candida albicans* BSI. ICU hospitalised
4	Candidemia	*Candida albicans* BSI. ICU hospitalised
5	Candidemia	*Candida albicans* BSI. ICU hospitalised
6	Candidemia	*Candida albicans* BSI. ICU hospitalised
7	Paracoccodioidomycosis	Positive direct examination and culture from cerebral abscess
8	Paracoccodioidomycosis	Positive culture from BAL
9	Cryptococcosis	Pulmonary cryptococcosis. HIV+ with positive *Cryptococcus* antigen in serum
10	Cryptococcosis	Immunodeficient with meningitis. Positive direct examination, culture and *Cryptococcus* antigen in CSF
11	*Trichosporonosis*	Immunodeficient (hematologic malignancy, and stem cell transplant). *Trichosporon asahii* BSI

Abbreviations: +, Positive; BAL, Bronchoalveolar lavage; BSI, Bloodstream infection; CSF, Cerebrospinal fluid; ICU, Intensive care unit.

## 4 DISCUSSION

This report describes the development and evaluation of the performance of the first lateral flow assay for the detection of anti‐*Sporothrix* antibodies. IMMY's *Sporothrix* antibody detection LFA is a rapid and accurate tool for addressing the diagnosis of sporotrichosis in humans. In addition, this LFA showed good performance for the diagnosis of atypical manifestations of CTS.

In this study, we identified individuals with a broad spectrum of clinical presentation, highlighting from this study the high prevalence of individuals with ocular CTS (13%). Ocular forms may occur due to contact with secretions of sick felines in the conjunctiva, demonstrating that zoonotic transmission has altered the observed profile of clinical manifestations.^8^, ^27^, ^28^, ^29^ We observed high sensitivity (92%) for the diagnosis of ocular CTS using the Anti‐Sporo LFA. The only ocular CTS false‐negative result was observed in a patient with early presentation of the disease (10 days since onset of symptoms).

Factors that can also affect sensitivity of antibody detection assays include host baseline conditions, such as patient's immunological status, age, the time of disease onset, the clinical form of the disease, fungal burden and exposure to antifungal treatment.^5^, ^27^, ^30^, ^31^, ^32^, ^33^ We found a statistically significant correlation between assay sensitivity and time of disease onset; being higher sensitivity in patients with more than 21 days of symptoms. Experimental studies in mice have demonstrated that detection of IgG antibodies appears after 14 days of infection, and in humans, it has already been observed that this can be longer than 21 days.^27^, ^32^ Ageing could be the factor that affects assay sensitivity.^24^, ^31^, ^34^ In this study, we observed a lower frequency of false negatives in children (12%) compared with adults (18%), but this was not statistically significant. To increase the chance of detecting cases, it is recommended to evaluate acute and convalescent specimens. This recommendation is based on the evaluation of seroconversion, which could add important evidence for the diagnosis of the disease.^35^ Since several factors influence the performance of Ab detection assays, results must be carefully analysed and compared with the clinical and epidemiological data.

Among the CTS false‐negative results, ten were lymphocutaneous CTS, six were fixed cutaneous CTS, and one was an ocular form. It is known that antibody circulation and immunoglobulin type could vary based on the clinical manifestation of the disease. In fixed cutaneous and lymphocutaneous forms, low concentrations of IgM and IgA have been reported.^5^, ^27^, ^30^ Therefore, negative results cannot exclude the presence of CTS.^31^ In addition, we observed two false‐negative results in patients with comorbidities. As previously reported, it is well known that the immune status of the host can affect the production of antibodies, affecting the performance of antibody detection assays.^17^

The Anti‐Sporo antibody LFA presented a global specificity of 82%. In the group of specimens from patients with other mycosis, we observed 11% cross‐reactions, this type of cross‐reaction with other fungal infections has been previously reported using other antibody detection assays.^31^, ^32^, ^36^, ^37^ Studies using an EIA anti‐*Sporothrix* detection assay reported false‐positive results with several fungi, including species of the genus *Histoplasma*, *Candida*, *Paracoccidioides* and *Cryptococcus*.^32^, ^37^, ^38^ Due to antigenic similarities of cell wall structures of some fungi, serological cross‐reactivity can occur, mainly due to glycosylated compounds.^37^, ^39^, ^40^ On the contrary, it is important to consider that the clinical and epidemiology of these infections are distinct. Correlation of patient risk factors could reduce the impact of false‐positive results.

We found in the group of apparently healthy volunteers (Group 3), a major number of false‐positive reactions (26%). None of these volunteers had epidemiological or clinical criteria for sporotrichosis. Additionally, most of these volunteers were healthcare workers residing in the endemic region for sporotrichosis. These characteristics would increase the risk of exposure to *Sporothrix* and other fungal pathogens able to produce cross‐reactions.^8^, ^19^, ^24^ Based on these findings, it is recommended to perform this test only in people who are symptomatic, or with a strong epidemiological link to CTS. In addition, future investigations focusing on product improvement are needed. These studies could be focused on the optimisation of antigen used for antibody detection, and the implementation of a LFA reader.

Study limitations are mainly related to lack of information of immune status of other infectious diseases from all patients and volunteers. Further studies are needed to know the performance of this assay when infections are caused by species of *Sporothrix* other than *S. brasiliensis*. In this study, specimens from cats and other species susceptible to develop sporotrichosis were not evaluated. In addition, it is necessary to evaluate this prototype in other sporotrichosis endemic regions.

There are some other assays for the detection of antibodies against *Sporothrix*. These assays are mainly based on EIA, latex agglutination, tube agglutination, complement fixation and indirect fluorescence. The main limitation of these assays is the limited commercial availability of kits.^34^, ^37^, ^41^ Currently, there is one kit available on the market for the detection of Ab; this kit uses a latex agglutination system (IMMY, Norman, USA). At the moment of writing this report, one laboratory in Brazil, BIDiagnostics, offers an EIA‐based assay for the detection of anti‐*Sporothrix* Ab. Limitations of this assay are the need to ship specimens, and the lack of in vitro diagnostics certification in human specimens.

The findings in this study suggest that IMMY's *Sporothrix* antibody detection LFA prototype is a promising tool for the diagnosis of CTS in humans. In addition, some major advantages of LFA technology are simplicity, rapid turn‐around for results, low cost, high accuracy, does not require complex laboratory infrastructure and personal training, and reagents could be transported and stored at room temperature. This test can improve the clinical suspicion of CTS, reducing time of therapy initiation and impacting the outbreak control.

## AUTHOR CONTRIBUTIONS

RC: Writing – original draft (lead); Conceptualisation (supporting); Investigation (lead); Methodology (supporting); Writing – review and editing (equal). KB: Methodology (lead); Conceptualisation (supporting); Writing – review and editing (equal). IW: Methodology (supporting); Writing – review and editing (equal). DHC: Writing – original draft (supporting); Conceptualisation (supporting); Investigation (supporting); Methodology (supporting); Writing – review and editing (equal). RD: Methodology (supporting); Conceptualisation (supporting); Writing – review and editing (equal). FQT: Conceptualisation (lead); Writing – original draft (supporting); Writing – review and editing (equal).

## FUNDING INFORMATION

This study was performed as part of a PhD program for the lead author (RC). Reagents were provided by IMMY (Norman, OK, USA). No external funding was received for this study.

## CONFLICT OF INTEREST

Regielly Cognialli, Izabella Weiss and Flavio Queiroz‐Telles declare no conflict of interest. Konner Bloss, Rachelle Davis and Diego H. Caceres are employees of IMMY. Reagents were provided by IMMY (Norman, OK, USA). No external funding was received for this study.

## Data Availability

The data that support the findings of this study are available from the corresponding author upon reasonable request.

## APPENDIX A

### Criteria for definition of human sporotrichosis. Summary of the Brazilian ministry of health guidelines

**Table:**

Case definition	Epidemiological criteria	Clinical criteria	Laboratory criteria
Possible	History of trauma or contact with sick cats	Suggestive lesions	Absent
Probable	History of trauma or contact with sick cats	Suggestive lesions	Veterinarian diagnosis: A – Microbiologic proved diagnosis by the veterinarian or veterinarian laboratory B ‐ Regional detection of feline cases from other sources: Zoonosis Control Centers, Mobile contacts to a reference centre, etc.
Proved	History of trauma or contact with sick cats	Suggestive lesions	Positive culture^a^ and/or histopathology^b^
Discarded	History of trauma or contact with sick cats	Suggestive lesions	Microbiologic and/or histopathologic proved diagnosis of another disease. Negative culture for *Sporothrix* spp.^c^

*Note*: Font: Adapted from‐ Guide to Health Surveillance (BRAZIL, 2021) and QUEIROZ‐TELLES et al.*,* 2022.

^a^ Standard method.

^b^ Histopathology can be non‐specific. The result must be analysed carefully and associated to clinical and epidemiology.

^c^ Only negative culture for *Sporothrix* spp. does not rule the diagnosis (limitation of the culture).
